# Biogenetic study of the emissions of species: *Pinus radiata*, *Eucalyptus globulus* Labill and *Alnus acuminata *in Riobamba canton, Ecuador

**DOI:** 10.12688/f1000research.19255.1

**Published:** 2019-07-05

**Authors:** Benito Mendoza, Marco Cruz, Lucero Carrera, Mauro Jimenez, Juan Caicedo, Miguel Osorio, Luis Santillán, Fabian Arias

**Affiliations:** 1 Universidad Nacional de Chimborazo, Riobamba, Ecuador; 2 Escuela Superior Politécnica de Chimborazo, Riobamba, Ecuador

**Keywords:** Biogenetic study, EMISSION, Pinus radiata, Eucalyptus globulus Labill, Alnus acuminata, Riobamba canton

## Abstract

**Background: **Air pollution is one of the biggest problems in the world, and it is generated by industrial production, vehicular flow and use of fossil fuels, leaving aside other important emission sources such as vegetation. The aim of this research is to quantify the emissions of natural volatile organic compounds produced by the forest species: *Eucalyptus globulus* L., *Pinus radiata* and *Alnus acuminata* in Riobamba, Ecuador.

**Methods: **Identification of plant coverings in the years 2014 and 2017was performed using geographic information systems tools, complemented with the application of the Guenther model for the calculation of monoterpenes and other organic volatile compounds; thus, to analyze the relationship between meteorological variables and concentrations of volatile organic compounds and nitrogen dioxide per species.

**Results: **Mathematical calculation of emissions in Riobamba showed that *Eucalyptus globulus* L. registered higher emissions in the years 2014-2017, followed by *Pinus radiata* and *Alnus acuminata*. These emissions are due to the vegetation cover covering each species. The analysis of volatile organic compounds in forest plantations in air is directly related to the emissions represented in the environment and correlated with the meteorological variables of temperature, global solar radiation and wind velocity. The proposed method manages to estimate concentrations of monoterpenes and volatile organic compounds for the two examined seasons, presenting the influence of the species introduced in this study such as *Eucalyptus globulus* L. and *Pinus radiata*, with a reduction in their emissions (less area found in the year 2017, with respect to 2014). However, the emission of *Alnus acuminata *can be quantified only in 2017, since in 2014 no records of this species were found.

**Conclusions:** Volatile organic compound concentrations in the air are directly related to the emissions represented spatially and correlated with the meteorological variables of temperature, global solar radiation and wind velocity.

## Introduction

The atmosphere contains many gases which, when presented in concentrations higher than normal, are poisonous to humans, animals and are harmful to plants; gases such as nitrogen oxides (NOx), sulphur (SOx), hydrocarbons, carbon monoxide (CO) and a wide variety of volatile organic compounds (VOCs) are considered primary pollutants, because they are emitted directly from a source. Secondary contaminants are formed by means of chemical reactions from the primary pollutants; ozone (O _3_) is found in this group ^1^. In recent years, Ecuador has been more interested in emissions of natural origin, giving rise to inventories of volatile organic compounds nationwide, obtaining 1,855,600 tons/year in 2010 ^2^. The Ministry of Environment, Ecuador (MEE) has developed emission inventories in the districts Ambato, Riobamba, Santo Domingo de los Colorados, Latacunga, Ibarra, Manta, Portoviejo, Esmeraldas and Milagro, giving a space to the biogenic emissions representing 3.3% of the total emissions in Riobamba ^3^. Riobamba is located at an altitude of 2750 m above sea leave; it is in the Sierra Central region and constitutes the capital of Chimborazo ^4^. The population of the rural areas of the Ecuadorian Highlands, including Riobamba, has been dedicated to agroforestry crops with commercial purposes ^5^. Some of these plant species are exotic, which in addition to causing negative effects to the soil, emit polluting gases that react in the atmosphere, giving rise to the formation of new compounds that may have negative effects on humans ^6^.

In this context, the objective of this study is to make an approximate quantification of the emissions of natural volatile organic compounds from the species *Pinus radiata*, *Eucalyptus globulus* L. and *Alnus acuminata* in the district, by the variation of plant coverings obtained based on spectral signatures, temperature analysis of the years 2014–2017 and application of the emission model proposed by Guenther.

## Methods

### Definition of monitoring plots

Based on the area occupied by each species, plots of circular form are arranged with an area of 500 m ^2^ each ^7^ applying the equation of finite populations to obtain the sample size ^8^:

$$n=\frac{Z^{2}\times p\times q\times N}{\left(N\times E^{2})+(Z^{2}\times p\times q\right)}\;(1)$$

Where: *n* represents the sample size; *Z*, 95% confidence level of = 1.96; *N*, study population; *E*, estimation error = 0.05; *p*, probability of success = 0.5; *q*, probability of failure = 0.5.

Sampling was carried out for 3 days (October 8, 9 and 10, 2018), 3750 spectral signatures of the three species under study were obtained with the Spectrum-Field Spec 4 radiometer, this in seven plots of *Eucalyptus globulus* L. four of *Pinus radiata* and four of *Alnus acuminata* ( Table 1).

**Table 1.** Description of the monitoring plots.

Species	Plot number	Vegetative state	Coordinates		
			x *	y *	Altitude, m
*Eucalyptus globulus* L.	1	Small trees	755647	9812023	3137
	2	High tress	755397	9811623	3195
	3	Small trees	756075	9811468	3155
	4	High tress	756031	9811558	3146
	5	High tress	755538	9811849	3115
	6	Small trees	755306	9811364	3250
	7	Small trees	755370	9811303	3220
*Pinus radiata*	1	Small trees	754935	9807413	3576
	2	Small trees	754995	9807455	3549
	3	High trees	755735	9807979	3453
	4	High trees	755779	9808061	3440
*Alnus acuminata*	1	Small trees	755796	9808122	3434
	2	Small trees	755720	9808059	3459
	3	Small trees	756155	9811455	3162
	4	Small trees	755386	9811688	3184

*Coordinates are expressed in Universal Transverse Mercator coordinate system (Hemisphere: South; Zone: 17).

The spectral signatures were treated statistically with SAMS 3.2 software; for the correction of jumps, the Jump Correction tool was used, which corrects the level of reflectance in the signature. The spectra that were found out of the trend of the vegetative states of the small trees (those up to 20 cm in diameter) and high trees (those exceeding 20 cm in diameter) of the three species under study, were eliminated with the help of the software Minitab 18, obtaining the standard deviation grouped to rule out significant differences between the spectra grouped by plots.

### Obtaining spectral signatures

The contact probe was used to analyze the spectral signatures of plants with the spectrum-radiometer Field Spec 4, selecting five samples distributed in a plot; each sample represents a tree, from which five leaf subsamples were taken from the canopy.

Spectral signatures were analyzed using View Spec Pro 6.2 and Minitab 18 software; the consistency of the spectral signature reflectance levels is also statistically verified using the SAMS software, discarding those that do not present a similar trend to the metadata group.

### Multitemporal study

The field assessment of the normalized difference vegetation index (NDVI) is calculated from the average wavelength between 640 to 670 nm and 850 to 880 nm, and in satellite images using bands 4 and 5 of the Landsat 8 Medi satellite to Equation 2 ^9^.

$$NDVI=\frac{NIR-R}{NIR+R}\;(2)$$

Where NIR is the atmospherically corrected reflectance corresponding to the near infrared and R is the atmospherically corrected reflectance corresponding to the red.

The spectral difference reflected in the NDVI is used for the comparison of forest species coverings in the years 2014–2017, obtained by a supervised classification with the maximum likelihood classifier algorithm, using as a basis Landsat 8 satellite images ^5, 6^ with a spatial resolution of 30 x 30 m per pixel. The satellite images were obtained from the portal of the United States Geological Survey (USGS) through the Global Visualization Viewer (GloVis), making a search of the satellite images of the study area (Riobamba-Ecuador) in the years 2014 and 2017 (with a maximum cloudiness of 25%), the satellite selected was Landsat 8 due to availability and good resolution per pixel. Once selected, each of the images was downloaded separately.

The calculation and the resulting maps are made with the rasterized calculator tool of the ArcGIS 10.3 software ^10^ ( QGIS is an open-access alternative), entering the bands 4 and 5 of the satellite images, extracting the values of the index of each pixel corresponding to the monitoring plots according to the vegetative state of the species.

To obtain the result, the maximum likelihood classification algorithm ^11^ is applied using ENVI 5.3 software. This is a comparison of the effects of the satellite image with those taken as training areas, thus assigning the pixels to the class to which they most likely belong. The resulting classification was exported to shapefile format.

### Temperature study

For the study of temperature, data from the automatic meteorological stations of: ESPOCH, UNACH, San Juan, Alao, Tunshi, Quimiag, and Urbina were used. In addition, to determine the hourly temperature, linear regression of the form *ax + b* was used ^12^. These data are interpolated with the universal kriging method ^13^, generating hourly temperature maps for the years 2014–2017 ^20^.

### Biogenic VOC (BVOC) emissions calculation

Emissions were calculated based on the temperature schedules generated for each month. It uses the biomass density values and emission factors for monoterpenes and BVOC proposed by Guenther, described in the *Underlying data*. Table 1.

### Monoterpenes

The time emissions of monoterpenes were calculated by means of the formulas posed by Guenther ^2^.

$$E_{mon}(K,\;time)=EF_{j}^{mon}\times M(T)\times FBD_{j}\times A\;(3)$$

Where E _mon_ (k, time) is hourly emission of monoterpenes in each *K* cell (µg/h), $EF_{j}^{mon}$ is standard emission factor of monoterpenes associated with *J* category soil use (µg/g.h), FBDj is density of foliar biomass of the *J* class of soil use (g/m ^2^), Ais area of each cell (900 m ^2^) and M(T): environmental correction factor belonging to the temperature ( Equation 4).

$$M(T)=exp\;(\beta .\;(T-T_{S}))\;(4)$$

Where: *β* is an empirical coefficient (0.09° *K* ^-1^); T is leaf temperature (equal to environmental temperature in °K); T _s_ is standard temperature (303 °K),

Daily emissions are obtained using Equation 5.

$$E_{mon}(K,\;daily)=\sum_{h=1}^{24}E_{mon}(k,\;hour)\;(5)$$

Monthly emissions are obtained using Equation 6.

$$E_{mon}(K,\;monthly)=30\times E_{mon}(k,\;daily)\;(6)$$

The calculation of the annual emissions of monoterpenes is obtained through Equation 7.

$$E_{mon}(K,\;annual)=\sum_{m=1}^{12}E_{mon}(k,\;monthly)\;(7)$$

### Other BVOC

These are calculated with Equation 8, which was also used previously for the calculation of monoterpenes, considering the variation of emission factors ^2^.

$$E_{BVOC}(K,\;time)=EF_{j}^{BVOC}\times M(T)\times FBD_{j}\times A\;(8)$$

Where: E _BVOC_ (k, time) is the hourly emission of BVOC in each *K* cell (µg/h) and $EF_{j}^{BVOC}$ is the standard emission factor of BVOC associated with the *J* category of soil use (µg/g.h).

Daily, monthly and annual emissions of other volatile organic compounds are obtained by Equation 5, Equation 6 and Equation 7, respectively.

### Measurement of volatile organic compounds (COV) and NO _2_

The experimental application consisted of measurements of VOC and NO _2_ concentrations, using the Aeroqual S-500 gas analyzer equipment between 11:00 and 15:00, due to the higher daily temperatures occurring in this range.

### Statistical analysis

The analysis of the existing correlation of the meteorological variables (temperature, solar radiation and wind velocity) with VOC concentrations was performed using Pearson's product-moment correlation coefficient ^14^. Two-way ANOVA with post hoc Turkey’s test were used for the statistical analysis of the NO _2_ concentrations, grouping the variables temperature and global solar radiation. For the graphical analysis, the moving average method of order 3 was applied to obtain a smoothing of the curves ^14^. In addition, the Pearson correlation method was used to assess the linear correlation between the VOC concentrations in the plantations of each plant species with the following variables: temperature, global solar radiation and wind speed. ^15^. Statistical analyses were performed using Minitab v18 (Minitab, Inc.).

## Results and discussion

### Evaluation of representative spectral signatures

In the spectral signature of *Eucalyptus globulus* L., the reflectance level in the vegetative states (sapling and timber) does not differ significantly in the range comprising the NDVI; the highest peak of the sapling state has a reflectance of 72.18% and a timber of 72.27% ^15^. This is due to the similarity in the structure of the leaves in the two vegetative states.

The spectral signature of *Pinus radiata* shows that the reflectance levels of the sapling state are slightly higher (83.82% in the highest peak), while in the timber the highest peak corresponds to 82.36% of reflectance.

In the spectral signature of *Alnus acuminata*, the representative spectral signature in the sapling state shows that the highest peak has 83.13% reflectance at wavelength 880 nm, which is located in the range comprising the NDVI.

### Comparative analysis of the NDVI

The NDVI values obtained in the field are elevated values approximated to 1, being dense and healthy vegetation ^16^. The highest value is presented in the sapling state of *Alnus acuminata* (0.887) and the lower result of the index corresponds to the species of *Pinus radiata* (0.795), whereas in the timber state, the highest value (0.808) corresponds to *Eucalyptus Globulus* L. and the lowest (0.819) to *Pinus radiata* (see *Underlying data*: Table 2).

Using the information from the NDVI maps of the satellite images, it was determined that in the year 2014 the minimum value (0.303) was of *Pinus radiata* in the timber state, and the highest in sapling state (0.622). In the year 2017, the highest value (0.537) corresponding to *Pinus radiata* in timber state and the lowest (0.384) is of *Alnus acuminata*.

### Variation of vegetal cover in the years 2014–2017

The plant coverings in the years 2014–2017 for the three forest species were obtained through a supervised classification, taking advantage of the spectral difference found in the NDVI values. The reliable results were verified by means of the maximum likelihood algorithm reflected in the confusion matrix, surpassing the value of 0.85 in the Kappa coefficient, considered an almost perfect classification according to Landi and Koch ^17^.

The variation in the area covered by forest species is important ( Figure 4, Figure 5), especially in *Eucalyptus globulus* L., which has suffered greater deforestation, and in the last three years it has decreased 469.22 ha, and *Pinus radiata* has reduced 228.11 ha in the same time. Since in the year 2014 no plantations were found; in 2017 there were 44.31 ha, located mainly in the Cacha parish due to the existing deforestation programs.

**Figure 1.**
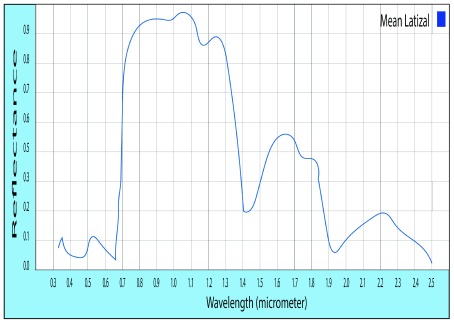
Standard of the spectral signature of *Eucalyptus globulus* L.

**Figure 2.**
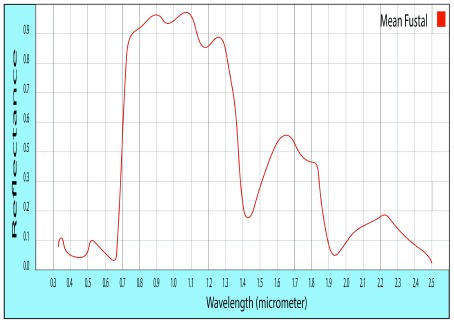
Standard of the spectral signature of *Pinus radiata*.

**Figure 3.**
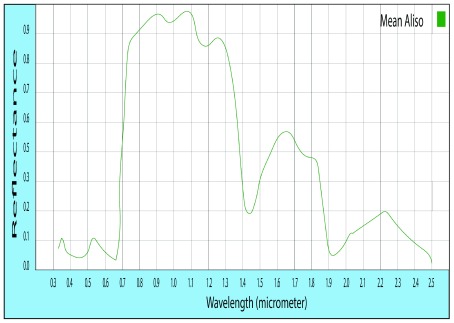
Standard of the spectral signature of *Alnus acuminata*.

**Figure 4.**
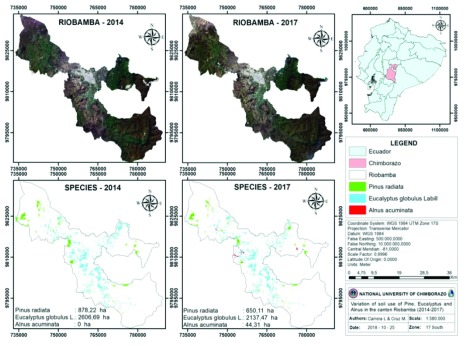
Variation of the soil use by forestry species in the years 2014–2017.

**Figure 5.**
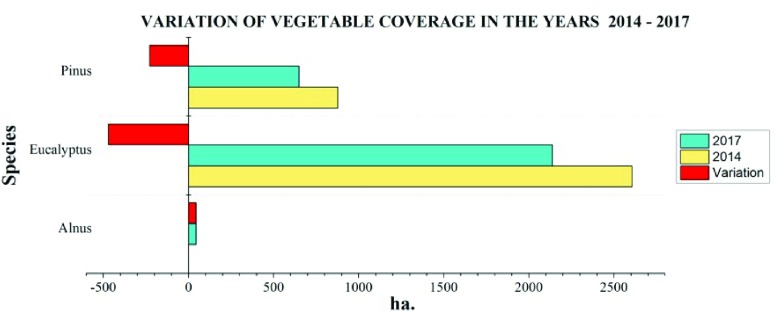
Variation of the vegetal cover in the years 2014–2017.

The annual gross deforestation in Riobamba shows that species planted for commercial purposes, such as *Pinus radiata*, contributes towards 76.04 ha/year of deforestation. *Eucalyptus globulus* L. is deforested by 156.41 ha/year, in contrast to *Alnus acuminata*, the area of which has increased by 14.77 ha/year. These values represent an important part of the average annual gross deforestation in Chimborazo ^16^, which reaches 928 ha/year.

### Temperature variations

**Figure 6.**
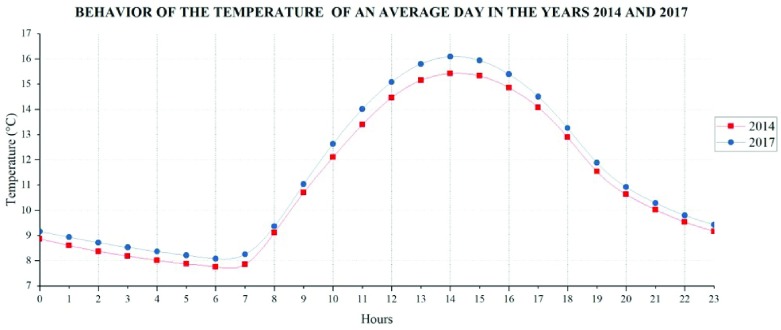
Time behavior of the average day temperature in 2014–2017.

The temperature variations with respect to time obtained from geostatistical analysis ( Figure 6) shows that in the year 2017 the average hourly temperatures are slightly higher than in the year 2014, emphasizing from 13:00 to 15:00 hours, time with the highest temperatures.

**Figure 7.**
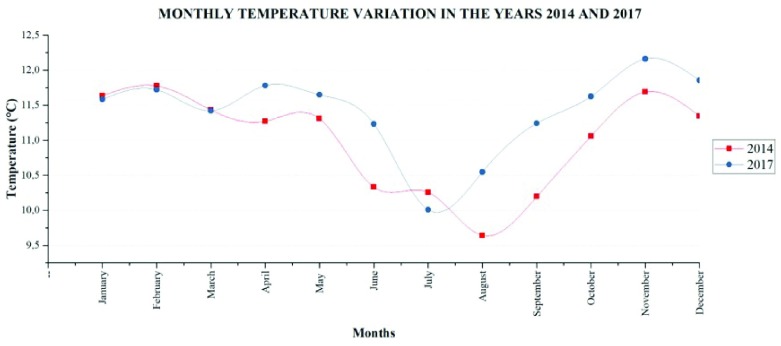
Variation of the average monthly temperature of the years 2014 and 2017.

Temperature behaves similarly in the two years ( Figure 7), but it is evident that in 2014 August is the month with the lowest average temperature, reaching 9.64°C, whereas, in 2017 July has the lowest average monthly temperature (10.01°C). The highest monthly average temperature values recorded in 2014 correspond to February (11.78°C); however, November 2017 has a higher value (12.16°C), thus, conditioning the emissions of natural VOC.

### Emissions of BVOC

**Figure 8.**
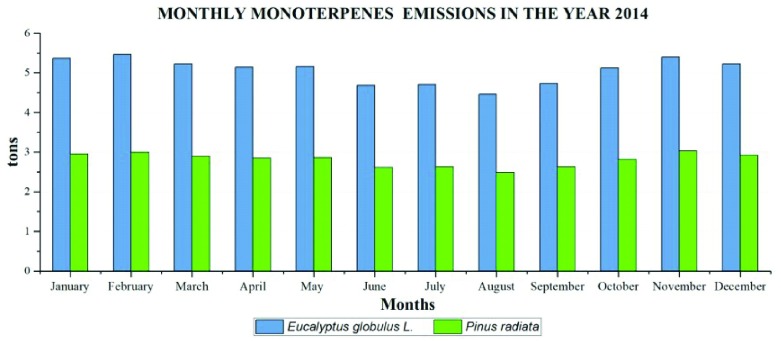
Monthly Emissions of Monoterpenes in the year 2014.

Monthly emissions of monoterpenes in the year 2014 were due in a greater proportion to the species of *Eucalyptus globulus* L. surpassing 5.40 tons, especially in February and November ( Figure 8). *Pinus radiata* emitted monoterpenes to a lesser amount, reaching maximum emissions of 3.03 tons in November. *Alnus acuminata* emissions were not recorded due to the absence of plantations.

The emissions of monoterpenes in 2014 of *Eucalyptus Globulus* L. correspond to 60.68 ton/year and 33.67 ton/year of *Pinus radiata*.

**Figure 9.**
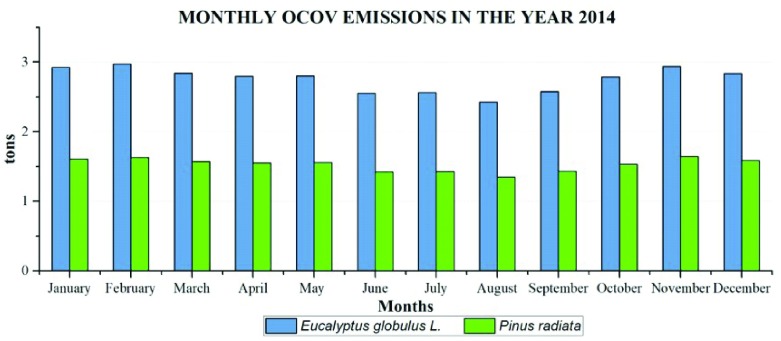
Monthly BVOC emissions in the year 2014.

Monthly emissions of BVOC in the year 2014 follow a similar annual pattern to monoterpenes. The emissions of *Eucalyptus globulus* L. and *Pinus Radiata* emit between 2.4 and 3 tons per month, reaching a maximum of 1.65 tons in November ( Figure 9). The total emissions of BVOC in 2014 by *Eucalyptus Globulus* L. were 33.01 ton/year and for *Pinus radiata* were 18.32 ton/year.

**Figure 10.**
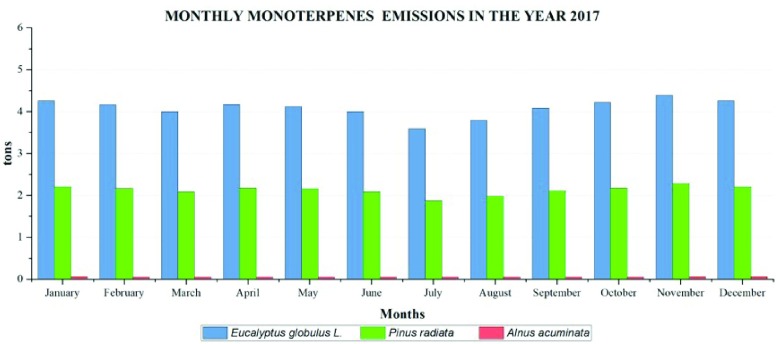
Monthly monoterpene emissions in the year 2017.

For monthly emissions of monoterpenes in the year 2017, the highest emissions are generated by *Eucalyptus globulus* L. ( Figure 10) and correspond to the month of November (4.39 tons). The lowest by *Eucalyptus globulus* L. occurred in July (3.59 tons). *Pinus radiata* emissions do not exceed 2.29 tons per month, evidencing that *Alnus acuminata* species presents extremely low emissions compared to the other species, reaching maximum emissions of 0.031 tons in November.

**Figure 11.**
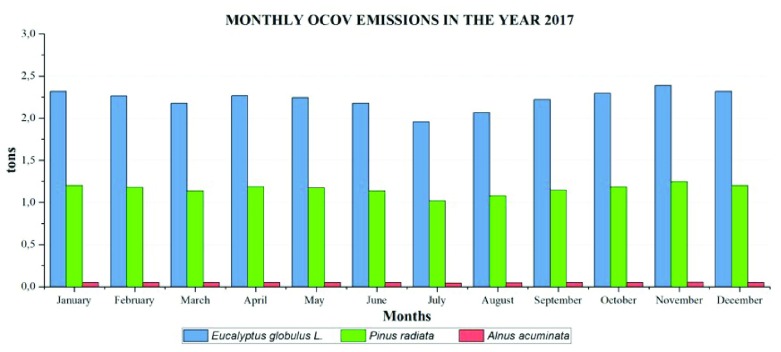
Monthly BVOC emissions in the year 2017.

Total emissions of monoterpenes in the 2017 were reduced due to the decreased vegetal cover of each species, presenting emissions of 49.05 ton/year for *Eucalyptus globulus* L., 25.49 ton/year for *Pinus radiata* and 0.035 ton/year for *Alnus acuminata*; only the latter has increased, since in 2014 it was not possible to find plantations.

Concerning monthly emissions of BVOC in the year 2017, the highest emissions for *Eucalyptus globulus* L. and *Pinus Radiata* occur in November (2.39 and 1.34 ton), and the lowest emissions were in July (1.95 and 1.02 ton). Emissions of *Alnus acuminata* have increased, reaching 0.051 tons in November and 0.41 in July, being the highest and lowest monthly emissions, respectively.

The total emissions of BVOC in 2017 in *Eucalyptus Globulus* L. was 26.29 ton/year and in *Pinus radiata* was 13.87 ton/year. These two species emit larger amounts of BVOC than *Alnus acuminata,* which only reached 0.571 ton/year.

### VOC concentrations in plantations of *Pinus radiata*

**Figure 12.**
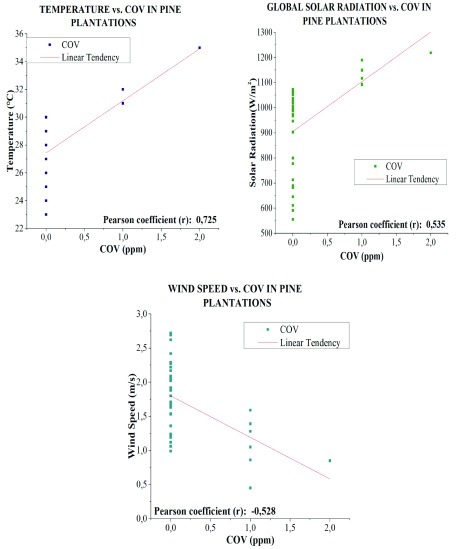
VOC Correlation and meteorological variables in *Pinus radiata*.

According to Pearson correlation coefficient analysis with a confidence level of 99%, VOC concentrations in plantations of *Pinus radiata*. have a positive significant linear correlation that is higher with temperature (R ^2^=0.725) and global solar radiation (R ^2^=0.535) ( Figure 12), indicating that as temperature and radiation increase, VOC emissions also increase.

Wind velocity has a significant negative linear correlation (R ^2^=0.528) with VOC emissions ( Figure 12), i.e., when wind velocity is lower, gases tend to accumulate in the planting area, thus increasing the concentration.

### VOC concentrations in plantations of *Eucalyptus globulus* L.

**Figure 13.**
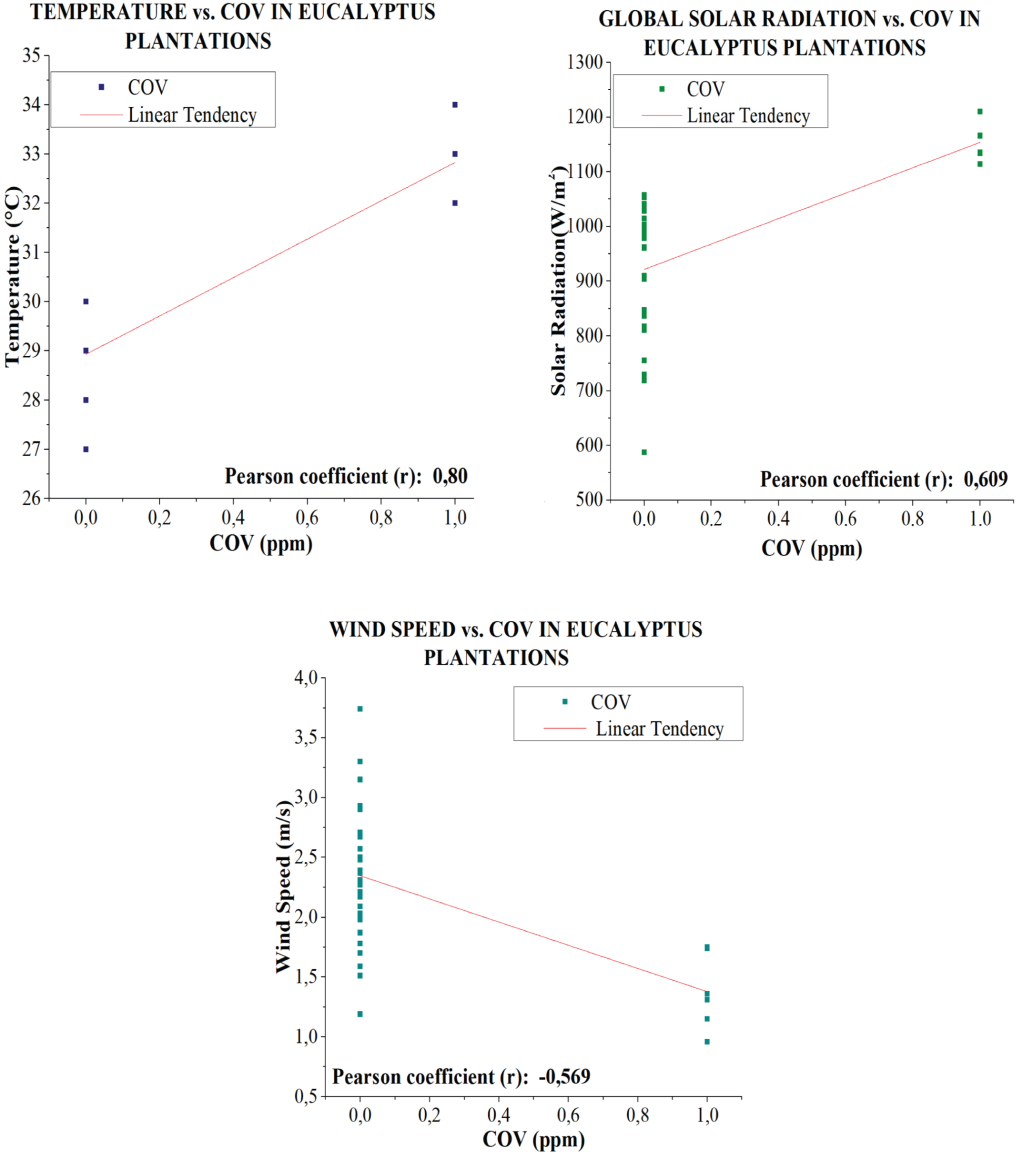
Correlation between VOC and meteorological variables in *Eucalyptus globulus* L.

VOC concentrations in plantations in *Eucalyptus globulus* L. show a positive significant linear correlation with temperature variables (R ^2^=0.80) and global solar radiation (R ^2^=0.609), and a significant negative linear correlation with wind velocity (R ^2^=-0.569) ( Figure 13).

The linear relationship between VOC and meteorological variables is lower in *Pinus radiata* compared to *Eucalyptus globulus* L., demonstrating a lesser influence of the meteorological variables on VOC emissions in *Pinus radiata* plantations.

### VOC concentrations in plantations of *Alnus acuminata*

Concentrations in the air were nil; this behavior can be related to the lower presence of existing biomass and low values of emission factors, especially monoterpenes.

### Analysis of NO _2 _concentrations

Two-way ANOVA showed that the average concentrations of NO _2_ do not differ from each other when related to the variables of temperature and solar radiation, in plantations of *Pinus radiata*, *Eucalyptus globulus* L, and *Alnus acuminata* ( *Underlying data*: Table 3).

The trend between the concentrations of NO _2_ and the variables temperature and global solar radiation is similar in plantations of *Pinus radiata*, *Eucalyptus globulus* L., and *Alnus acuminata*, demonstrating the relationship between the behavior of the climatic variables and NO _2_ concentrations in an environment with low anthropogenic intervention.

## Conclusions

The representative spectral signatures of each species were obtained. The reflectance values were similar in the vegetative and timber states, allowing the generalization in each species, finding that the maximum level of reflectance in *Eucalyptus Globulus* L. is 72.2%, in *Pinus radiata* is 83.8% and in *Alnus acuminata* is 83.1%. The spectral difference found among the species allowed the obtaining of the NDVI vegetation index, which served as a basis for an optimum dissolution among classes, identifying the exact geographical location of each plant species.

Temperature determines the emission of volatile organic natural compounds, particularly between the time range between 13:00 and 15:00. The spatial distribution of the temperature with respect to time indicated that biogenic emissions are concentrated in the central area of the parish, depending on the presence of forest plantations. In the year 2014, February was the month with higher temperatures reaching an average of 11.78°C, whereas, in the year 2017 the highest average temperature reached is 12.16°C in November. The lower average temperatures in 2014 was August (9.64°C) and in 2017 was July (10.01°C).

Emissions of monoterpenes by *Eucalyptus globulus* L. in 2014 were 60.68 ton/year and were 49.05 ton/year in 2017. Emissions of BVOC were 33.01 tons in 2014 and 26.29 tons in 2017. *Pinus radiata* emitted 33.67 ton/year of monoterpenes in 2014 and 25.49 ton/year in 2017. Emissions of BVOC in 2014 were 18.32 ton/year, and were 13.87 ton/year in 2017. In 2017, *Alnus acuminata* emitted 0.035 tons/year of monoterpenes and 0.571 tons/year of BVOC. *Eucalyptus globulus* L. is the species with the highest emissions in both years due to the greater number of plantations, followed by *Pinus radiata* and *Alnus acuminata*. At the general level, *Eucalyptus globulus* L. and *Pinus radiata* record a decrease in emissions in 2017 when compared with 2014, which is linked to deforestation; unlike *Alnus acuminata*, which exhibited a small increase in plantations due to existing reforestation plans, so increased in emissions in the same way.

## Data availability

### Underlying data

Figshare: Raw data for Biogenetic study of the emissions of species: Pinus radiata, Eucalyptus globulus Labill and Alnus acuminata in Riobamba canton, Ecuador, https://doi.org/10.6084/m9.figshare.8081216.v1 ^21^

This project contains the following underlying data:

- Spreadsheet containing raw toxicity data for biomass density and emission factors for monoterpenes and OCOV (Table 1), calculated values of the Normalized difference vegetation index (Table 2) and analysis of variance for NO _2_ concentrations (Table 3)

Figshare: Raw data for NVDI calculation, https://doi.org/10.6084/m9.figshare.8323670.v1 ^22^

Figshare: Temperatures for each plot and each month, https://doi.org/10.6084/m9.figshare.8323688.v1 ^23^

Figshare: Monoterpenes/BVOC, https://doi.org/10.6084/m9.figshare.8323715.v1 ^24^

Figshare: Categories of Soil Use, https://doi.org/10.6084/m9.figshare.8323799.v1 ^25^

Figshare: VOC and NO2 levels for each plot, https://doi.org/10.6084/m9.figshare.8323832.v1 ^26^

Data are available under the terms of the Creative Commons Attribution 4.0 International license (CC-BY 4.0).
