# Supplementary material for: Biogenetic study of the emissions of species: Pinus radiata, Eucalyptus globulus Labill and Alnus acuminata in Riobamba canton, Ecuador
Source: F1000Res. 2019 Jul 5;8:1012. [Version 1] doi: 10.12688/f1000research.19255.1 (PMC6852338; doi:10.12688/f1000research.19255.1)
Supplement: Peer review history

# Peer review: Reviewer response for version 1

**DOI:** 10.5256/f1000research.21103.r56026

**Published:** 2019-11-12

**Authors:** Holger Benavides-Muñoz

**Affiliations:** 1 Universidad Técnica Particular de Loja (UTPL), Loja, Ecuador

**General comments:**

 The article is within the scope of the F1000 Research Open for Science.

 The abstract clearly explains the goal and the methodology as well as the flow of the paper. The method is well defined and well-illustrated as it is. The authors are using modern concepts, pertinent equations and numerical comparisons between relevant variables which are clear to understand; besides, the authors detailed the data validation process with tools for spectral signatures.

 Although the authors quantify the emissions of natural volatile organic compounds produced by the forest species: *Eucalyptus globulus* L., *Pinus radiata* and *Alnus acuminata* in Riobamba, Ecuador, in the "Results and Discussions" the authors need to discuss the limitations of this study.

**Specific comments** (Please make corresponding adjustments):

1. In Table 1, lines: 2, 4 y 5 the terms “High tress” should be replaced by “High trees”.
2. The text included in all figures should be improved with more resolution and with uniform font sizes.
3. On page 5, “Measurement of volatile organic compounds (COV) and NO _2_” should be replaced by “Measurement of volatile organic compounds (VOC) and NO _2_”.
4. On page 9, figure 9 “OCOV” should be replaced by “OVOC” or BVOC?
5. On page 11, figure 11 “OCOV” should be replaced by “OVOC” or BVOC?
6. On page 11, figures 12 “COV” should be replaced by “VOC”.
7. On page 12, figures 13 “COV” should be replaced by “VOC”.
8. On page 13, “… **OCOV** (Table 1)” must be confirmed if the authors refer to “other volatile organic compounds **OVOC**”, or “oxygenated volatile organic compounds **OVOC**” or BVOC? Please make corresponding adjustments.
9. Recommendation: The authors should include the following references:
  - Préndez, M., Araya, M., Criollo, C., Egas, C., Farías, I., Fuentealba, R., & González, E. (2019). Urban Trees and Their Relationship with Air Pollution by Particulate Matter and Ozone in Santiago, Chile. In Urban Climates in Latin America (pp. 167-206). Springer, Cham. ^1^
  - González-García, S., Ferro, F. S., Silva, D. A. L., Feijoo, G., Lahr, F. A. R., & Moreira, M. T. (2019). Cross-country comparison on environmental impacts of particleboard production in Brazil and Spain. Resources, Conservation and Recycling, 150, 104434. ^2^

I confirm that I have read this submission and believe that I have an appropriate level of expertise to confirm that it is of an acceptable scientific standard.

## References

[1] : Urban Trees and Their Relationship with Air Pollution by Particulate Matter and Ozone in Santiago, Chile.2019; 10.1007/978-3-319-97013-4_8 167-206 10.1007/978-3-319-97013-4_8

[2] : Cross-country comparison on environmental impacts of particleboard production in Brazil and Spain. *Resources, Conservation and Recycling*.2019;150: 10.1016/j.resconrec.2019.104434 10.1016/j.resconrec.2019.104434

# Peer review: Reviewer response for version 1

**DOI:** 10.5256/f1000research.21103.r51402

**Published:** 2019-08-20

**Authors:** Mufreh S. Al-Rashidi

**Affiliations:** 1 Crisis Decision Support Program, Environment and Life Sciences Research Center, Kuwait Institute for Scientific Research, Kuwait City, Kuwait

The article addresses important natural volatile organic compounds (NVOC) sources of emissions to atmosphere from a three biogenic forest species, the *Eucalyptus globulus*, *Pinus radiata* and *Alnus acuminata* in Riobamba, Ecuador as a case study. The research methodology is well defined using different research tools linked with each other’s to provide the results. The study conclusions are practical and inline with the results finding.

 However, the following minor comments and changes need to be taken into consideration by the authors:

1. It is better in the titles of the article the word “Biogenetic study” change to “ Biogenic study” as the word Biogenic signified for the emission of substance (i.e., VOC, BVOC and NO2) from a living material such the plants and/or species in this study.
2. In introduction, 2 ^nd^ line, “as nitrogen oxides (NOx), sulphur (SOx), …” to be change to  “as nitrogen oxides (NOx), sulphur oxides (SOx), …”.
3. Figures 1, 2, 3, and 4 need to be reproduced with high resolution ones. In Figures 12 and 13, the x-axis name should be change from “COV (ppm)” to “VOC (ppm)”.
4. Most of the references are documented research and/or study carried out in Ecuador in Spanish language, authors recommended to update the references section with the original source of references which are mostly published in English such as the Guenther model these references are as follows:
  - Guenther, A., Monson, R. K., and Fall, R.: Isoprene and monoterpene emission rate variability: Observations with Eucalyptus and emission rate algorithm development, J. Geophys. Res., 96, 10799–10808, 1991 ^1^.
  - Guenther, A., Hewitt, C. N., Erickson, D., Fall, R., Geron, C., Graedel, T., Harley, P., Klinger, L., Lerdau, M., McKay, W. A., Pierce, T., Scholes, B., Steinbrecher, R., Tallamraju, R., Taylor, J., and Zimmermann, P.: A global model of natural volatile organic compound emissions, J. Geophys. Res., 100, 8873–8892, 1995 ^2^.
  - Guenther, A., Karl, T., Harley, P., Wiedinmyer, C., Palmer, P. I., and Geron, C.: Estimates of global terrestrial isoprene emissions using MEGAN (Model of Emissions of Gases and Aerosols from Nature), Atmos. Chem. Phys., 6, 3181-3210, doi:10.5194/acp-6- 3181-2006, 2006 ^3^.

I confirm that I have read this submission and believe that I have an appropriate level of expertise to confirm that it is of an acceptable scientific standard.

## References

[1] : Isoprene and monoterpene emission rate variability: Observations with eucalyptus and emission rate algorithm development. *Journal of Geophysical Research*.1991;96(D6) : 10.1029/91JD00960 10.1029/91JD00960

[2] : A global model of natural volatile organic compound emissions. *Journal of Geophysical Research*.1995;100(D5) : 10.1029/94JD02950 10.1029/94JD02950

[3] : Estimates of global terrestrial isoprene emissions using MEGAN (Model of Emissions of Gases and Aerosols from Nature). *Atmospheric Chemistry and Physics*.2006;6(11) : 10.5194/acp-6-3181-2006 3181-3210 10.5194/acp-6-3181-2006
